# Asymmetrical hybridization and gene flow between *Eisenia andrei* and *E*. *fetida* lumbricid earthworms

**DOI:** 10.1371/journal.pone.0204469

**Published:** 2018-09-21

**Authors:** Barbara Plytycz, Janusz Bigaj, Tomasz Panz, Paweł Grzmil

**Affiliations:** 1 Department of Evolutionary Immunology, Institute of Zoology and Biomedical Research, Jagiellonian University, Krakow, Poland; 2 Faculty of Biochemistry, Biophysics and Biotechnology, Jagiellonian University, Krakow, Poland; 3 Department of Genetics and Evolution, Institute of Zoology and Biomedical Research, Jagiellonian University, Krakow, Poland; University of Delhi, INDIA

## Abstract

Uniformly pigmented *Eisenia andrei* (Ea) and striped *E*. *fetida* (Ef) lumbricid earthworms are hermaphrodites capable of self-fertilization, cross-fertilization, and asymmetrical hybridization. The latter was detected by genotyping of F1 and F2 progeny of the controlled Ea+Ef pairs by species-specific sequences of maternal mitochondrial COI genes and maternal/paternal nuclear S28 rRNA genes. Among F1offspring there were self-fertilized Ea (aAA), Ef (fFF), and cross-fertilized fertile Ea-derived hybrids (aAF); the latter mated with Ea and gave new generation of Ea and hybrids, while mated with Ef gave Ea, Ef, Ea-derived hybrids and sterile Ef-derived hybrids (fFA). Coelomic fluid of Ea exhibits unique fluorescence spectra called here the M-fluorescence considered as a molecular biomarker of this species. Since similar fluorescence was detected also in some Ef (hypothetical hybrids?), the aim of present investigations was to identify the M-positive earthworms among families genotyped previously. It was assumed that factor/s responsible for metabolic pathways leading to production of undefined yet M-fluorophore might be encoded/controlled by alleles of hypothetical nuclear gene of *Eisenia* sp. segregating independently from species-specific S28 rRNA nuclear genes, where ‘MM’ or ‘Mm’ alleles determine M-positivity while ‘mm’ alleles determine M-negative phenotypes. Spectra of M-fluorescence were detected in all 10 Ea (aAAMM) and 19 Ea-derived hybrids (aAFMm), three of four Ef-derived hybrids (fFAMm) and one ‘atypical’ Ef (fFFMm) among 13 Ef earthworms. Among progeny of ‘atypical’ M-positive Ef (fFFMm) reappeared ‘typical’ M-negative Ef (fFFmm), confirming such hypothesis. Alternatively, the M-fluorescence might be dependent on unknown gene products of vertically-transmitted *Ea*-specific symbiotic bacteria sexually transferred to the Ef partner. Hypotheses of intrinsic and external origin of M-fluorescence might complement each other. The presence/absence of M-fluorophore does not correspond with body pigmentation patterns; Ef-characteristic banding appeared in posterior parts of hybrids body. In conclusion, Ea/Ef hybridization may serve for further studies on bi-directional gene flow.

## Introduction

Lumbricid earthworms from *Eisenia* sp. are valuable models in various scientific disciplines like biochemistry, ecotoxicology, and biomedicine [1–5] where proper species delimitation is crucial. This concern mainly uniformly pigmented *Eisenia andrei* (Ea) and striped *E*. *fetida* (Ef), originally described as pigmentation morphs of the one species spelled as *Eisenia foetida*, then as its two subspecies, and later on as two independent species with reproductive barrier [6] forming two distinct clades on phylogenetic tree based on species-specific DNA sequences [7–9].

Body pigmentation is often not conclusive, thus during our earlier studies of Ea/Ef delivered from France we have used various methods for proper distinction of specimens of these two species [10]; among others, coelomic fluid was analyzed in respect of presence of fluorescence spectra considered to be a fingerprint of *E*. *andrei*, hypothetically derived from 4-methylumbelliferyl β-D-glucoronide [11], called the MUG fluorophore [10; 12; 13], and here shortly the M-fluorophore. Contrary to our expectation, we have detected such fluorescent biomarker not exclusively in Ea but also in some Ef specimens–thus we considered them as hypothetical hybrids [10; 12]. Just this observation, together with a wide spectrum of pigmentation patterns of earthworms from our Ea/Ef cultures prompted us to test a hypothesis about the existence of inter-specific hybrids between Ea and Ef, both of them being simultaneous hermaphrodites [14] capable to self-fertilization [15].

Hybridization was detected by genotyping of F1 and F2 progeny of the controlled Ea+Ef pairs by species-specific sequences of both haploid mitochondrial COI genes of maternal origin [16; 17] (‘a’ or ‘f’ for Ea or Ef, respectively) and diploid nuclear 28S rRNA genes of maternal/paternal origin (either ‘A’ for Ea or ‘F’ for Ef). Among F1offspring there were self-fertilized Ea (aAA), Ef (fFF), and cross-fertilized fertile hybrids (aAF) derived from Ea ova; the aAF hybrids mated with Ea gave new generation of Ea and hybrids, and while mating with Ef gave Ea, Ef, aAF and sterile fFA hybrids derived from Ef ova. Using the methods of the combined mitochondrial and nuclear markers we detected on the Ea branch of the COI-based phylogram both the ‘pure’ Ea specimens (aAA) and relatively common inter-specific hybrids (aAF), while on the Ef branch there were both ‘pure’ fFF specimens and a few sterile fFA hybrids [18].

Since earthworm genotyping was performed on DNA extracted from amputated (and then regenerating [19]) tail tips, the same parental, F1, and F2 earthworms served as donors of coelomic fluid (that was gradually restored [12; 13]) for analysis in respect of presence/absence of M-fluorophore. The aim of such analyses was answering the question how molecular marker specific for *E*. *andrei* could be transferred to some *E*. *fetida* earthworms?

Hypothetically, the M-fluorescence might be dependent either on the metabolic pathway/s of *Eisenia* sp. itself, or might be derived from vertically transmitted *E*. *andrei*-specific symbiotic bacteria that can ‘infect’ partners of copulation. The results of tracking the M-positive earthworms within their families from previous investigations were consistent with hypothesis of the intrinsic origin of fluorophore; the dominant M-allele might be transmitted from M-positive Ea (aAAMM) to fertile Ea-derived M-positive hybrids (aAFMm) and then to ‘atypical’ M-positive Ef (fFFmM) earthworm and sterile Ef-derived hybrids (fFAmM). Such intrinsic pathway was also consistent with reappearance of M-negative Ef (fFFmm) earthworms in long-lasting cultures of atypical M-positive Ef (fFFMm). However, hypothetical participation of microbiome-derived factors in production of M-fluorophore cannot be neglected. The presence/absence of M-fluorophore does not correspond with body pigmentation pattern.

## Materials and methods

### Experimental animals

Adult composting earthworms *Eisenia andrei* (Ea) and *Eisenia fetida* (Ef) from laboratory colonies at the Lille University (France) were reared for generations in the Institute of Zoology and Biomedical Research of the Jagiellonian University, Krakow, Poland.

The main analyses of coelomic fluid were performed on 46 out of 158 descendants of laboratory-paired M-positive Ea and M-negative Ef specimens genotyped previously [18]. In short, during previous investigations the pairs of freshly hatched earthworms were cultured until cocoon production/reproduction. Supravitally amputated tail tips of these parental specimens and their offspring served as a source of individually numbered DNA samples genetically analyzed in two ways: 1) by species-specific (maternally derived) haploid mitochondrial DNA sequences of the COI gene being either ‘a’ for worms from Ea ova or ‘f’ for worms from Ef ova; 2) by the diploid maternal/paternal species-specific (A for Ea and F for Ef) nuclear DNA sequences of 28S ribosomal gene. The description of genotypes were as follow: ‘aAA’ for Ea, ‘fFF’ for Ef, and aAF or fFA for their hybrids derived either from the ‘aA’ or ‘fF’ ova, respectively. Among offspring of Ea+Ef pairs there were mainly aAA and fFF earthworms resulted from the facilitated self-fertilization and some aAF hybrids from aA ova but none fFA hybrids from fF ova. The aAF hybrids mated with Ea gave a new generation of Ea and aAF hybrids, while mated with fFF gave fFF, aAF, and sterile fFA hybrids. Pairs of hybrids, both aAF and fFA, produced plenty cocoons but no hatchlings [18].

Proof-of-concept investigations were performed on coelomic fluid of specimens from long-lasting cultures of M-positive Ea (EaMp), ‘typical’ M-negative Ef (EfMn), and ‘atypical’ M-positive Ef (EfMp). The EfMp individuals were identified in 2013 during our previous studies [10].

Pigmentation patterns were photographically documented with the DSL camera (Sony SLT-A58).

### Analysis of M-fluorescence in coelomocyte-containing coelomic fluid

For main experiments, 46 genetically identified aAA, fFF, aAF, or fFA specimens from previous study [18] of similar body weights (X = 0.77+0.18 g), were used for analyses of coelomic fluid.

Spectrofluorimetric analysis of the M-fluorophore in non-invasively retrieved coelomic fluid was performed by slightly modified method described previously [10; 12; 13]. After overnight depuration on moist filter papers, earthworm were immersed in 3 mL 0.9% Natrium chloratum (Kutno, Poland) and electrostimulated for 30 sec with a mild electric current (4.5V) for coelomic fluid extrusion through dorsal pores during animal body movements. After fluid extrusion the earthworms were returned to their original boxes. One mL of the extruded coelomocyte-containing coelomic fluid was supplemented with 20uL of Triton (Sigma-Aldrich) and shaked for 20 min on Elpon Laboratory Shaker type 358S to dissolve cellular components. Then samples were adjusted with PBS to 2 mL and final 1% Triton lysates were analyzed using Perkin-Elmer Spectrofluorimeter LS50B. As previously [10; 12; 13], emission spectra of M-fluorophore were recorded between 340 and 480 nm (lambda at 320 nm, peak at 380 nm) while excitation spectra between 260 and 360 nm (lambda at 380 nm, peak at 320 nm). Fluorophores are gradually restored in coelomic fluid of electrostimulated arthworms [12; 13] thus–when necessary–the procedure of coelomic fluid extrusion/analysis was repeated for the same specimens after earthworms’ 4-week recovery in soil.

## Results

### M-positive and M-negative specimens among genotyped *Eisenia* sp. earthworms

**Fig 1:**
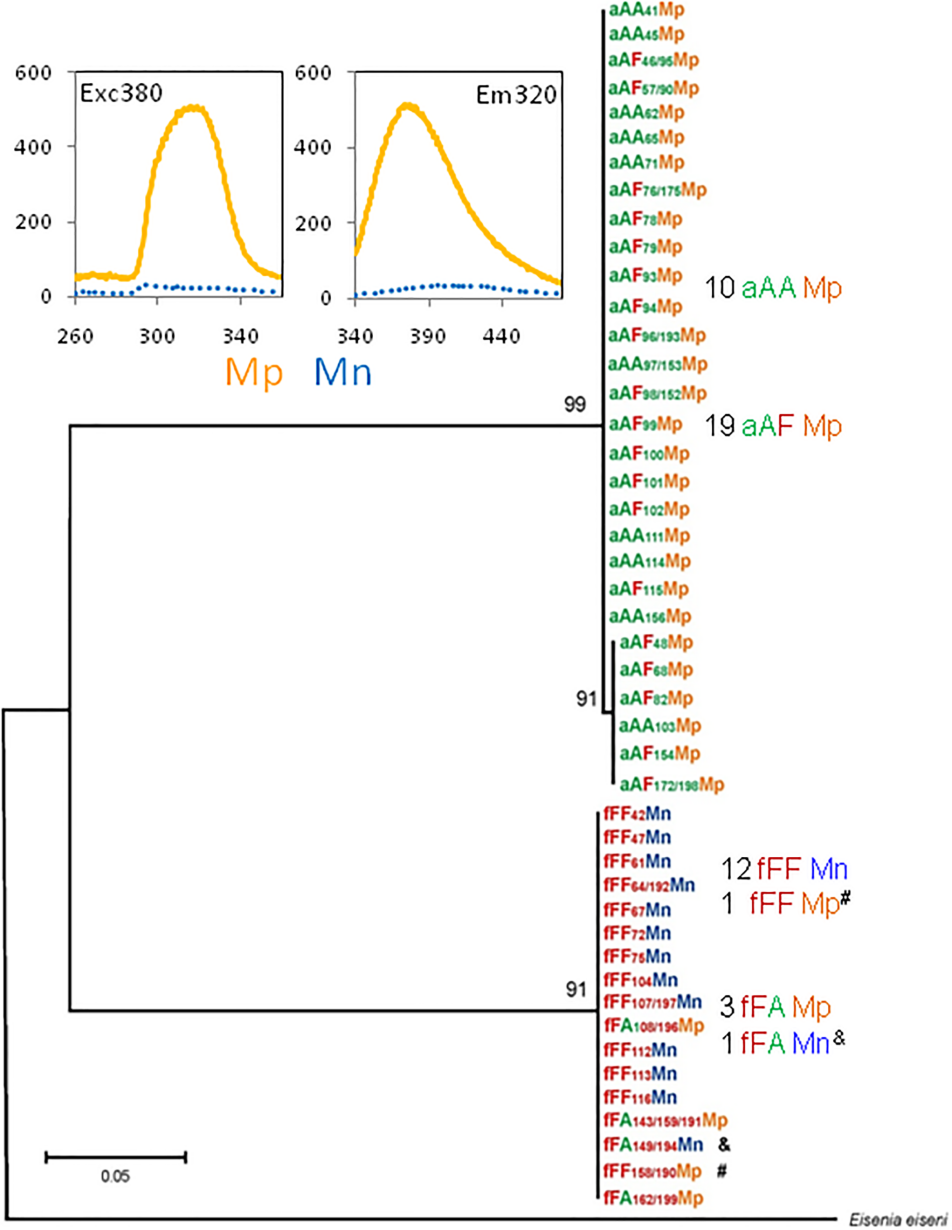
Fluorescence spectra of M-fluorophore and its presence/absence on phylogram of Ea, Ef, and their hybrids. The maximum-likelihood phylogram constructed according to sequences of the maternal mitochondrial COI gene (‘a’ or ‘f’) of *Ea/Ef* individually coded earthworms characterized also by sequences of their nucler 28S rRNA genes (‘AA’, ‘fFF’, ‘aAF’ or ‘fFA’) and phenotypes of their M-fluorophore as M-positive (Mp) or M-negative (Mn). &: atypical Mug-negative hybrid fFA_149/194_Mn; #: atypical MUG-positive specimen fFF_158/190_. Genebank accession numbers are given in [18]. Inset: Examples of fluorescence spectra of excitation (left) and emission (right) in coelomic fluid of Mp (orange solid lines) and Mn (blue dotted lines) specimens.

The M-positive (Mp) earthworms exhibited distinct spectra of fluorescence with a peak of absorbance at 314–320 nm (λ = 380) and a peak of emission at 370–380 nm (λ = 320), while the M-negative (Mn) earthworms were devoid of such fluorescence in Triton-lysates of coelomic fluid (Inset in Fig 1). As visible on phylogenetic tree of 46 descendants of Ea+Ef earthworms arranged on the basis of mitochondrial COI gene of maternal origin, all 29 specimens derived from *E*. *andrei* ova, i.e. 10 aAA and19 aAF hybrids, were M-positive. Thirteen specimens from *E*. *fetida* ova were M-negative, among them 12 fFF earthworm and one fFA hybrid; only one fFF specimen and three fFA hybrids were M-positive (Fig 1).

### Genealogy of M-positive and M-negative earthworms

**Fig 2:**
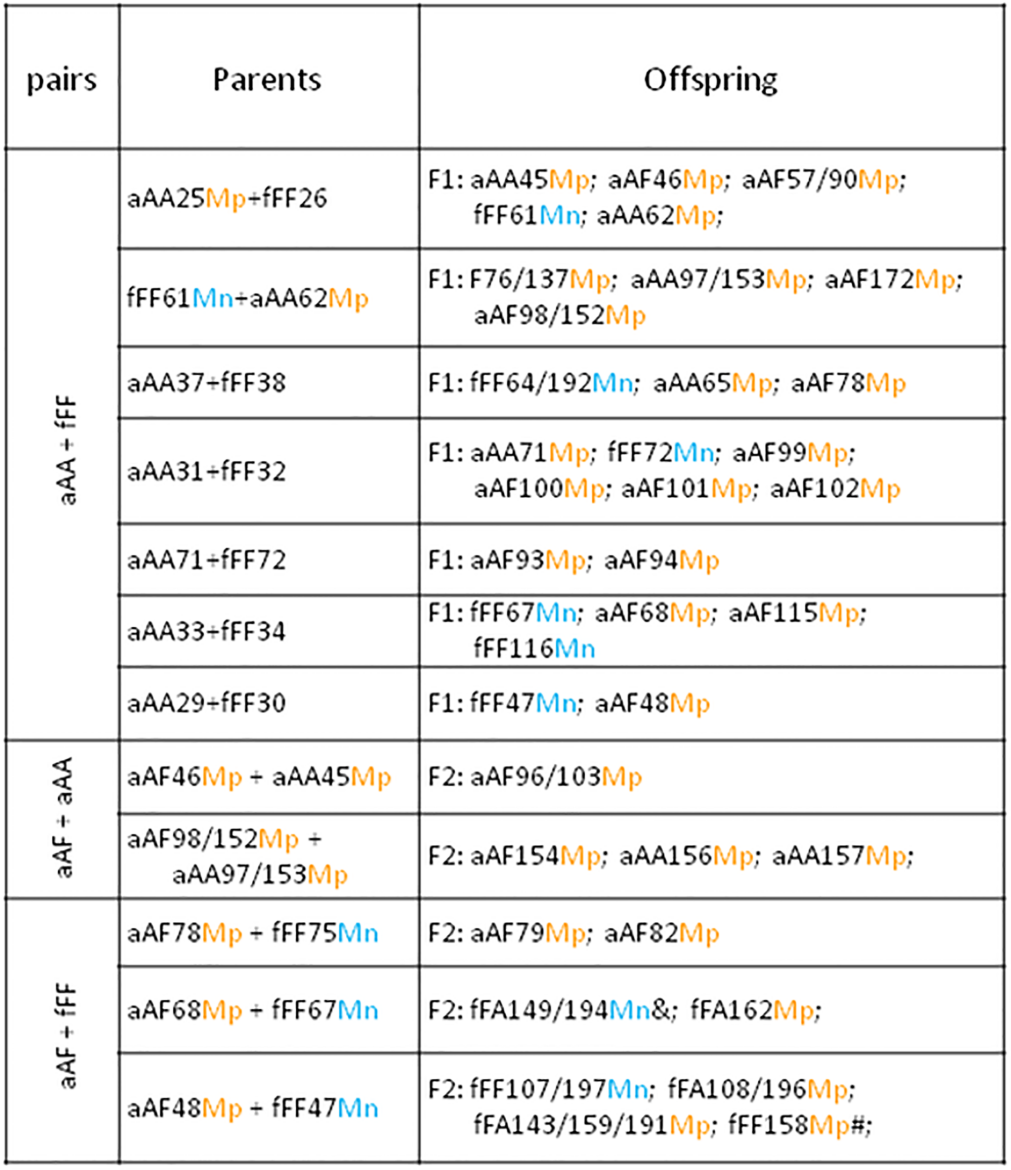
Relationships within families of Ea, Ef and their hybrids with and without the M-fluorophore. Progeny of Ea and Ef parental species (aAA+fFF), and crosses between aAF hybrids and Ea (aAF+aAA) or Ef (aAF+fFF) earthworms. Coded specimens are either M-positive (Mp) or M-negative (Mn). Symbols are the same as on Fig 1.

Genealogy of M-positive and M-negative descendants of Ea+Ef pairs has been shown on Fig 2. Among F1 offspring of pairs of parental specimens Ea+Ef there are M-positive Ea, M-negative Ef, and M-positive aAF hybrids from Ea ova, but none fFA hybrid from the Ef ova. The aAFMp hybrids paired with aAAMp specimens gave F2 generation of aAAMp pure Ea specimens and aAFMp hybrids. The aAFMp hybrids paired with M-negative fFF earthworms gave four kinds of F2 specimens, i.e. ‘typical’ fFFMn earthworms, one M-positive Ef earthworm (fFFMp) and also four hybrids from Ef ova, of which three were M-positive (fFAMp) and one was M-negative (fFAMn) (Fig 2).

### Speculations on genotypes of M-positive and M-negative *Eisenia* sp. earthworms

Hypothetically the factor/s responsible for M-fluorescence might be encoded/controlled by the nuclear dominant ‘M’ allele of some unknown gene/s of *Eisenia* sp. while two recessive ‘mm’ alleles determine M-negative phenotype. Thus genotypes of phenotypically M-positive earthworms are either of MM or Mm, while genotypes of M-negative specimens are always ‘mm’.

Hypothetically, M/m alleles segregate independently from the nuclear A/F sequences of 28S rRNA gene. Therefore the genotype of M-positive Ea specimens may be either aAAMM or aAAMm, while the genotype of M-negative Ef specimens may be only fFFmm. Inter-specific hybrids might be either aAFMm/aAFmM or fFAMm/fFAmM, with the first written allele of each gene being of maternal origin, while Mm/mM have the same phenotypic effects.

**Fig 3:**
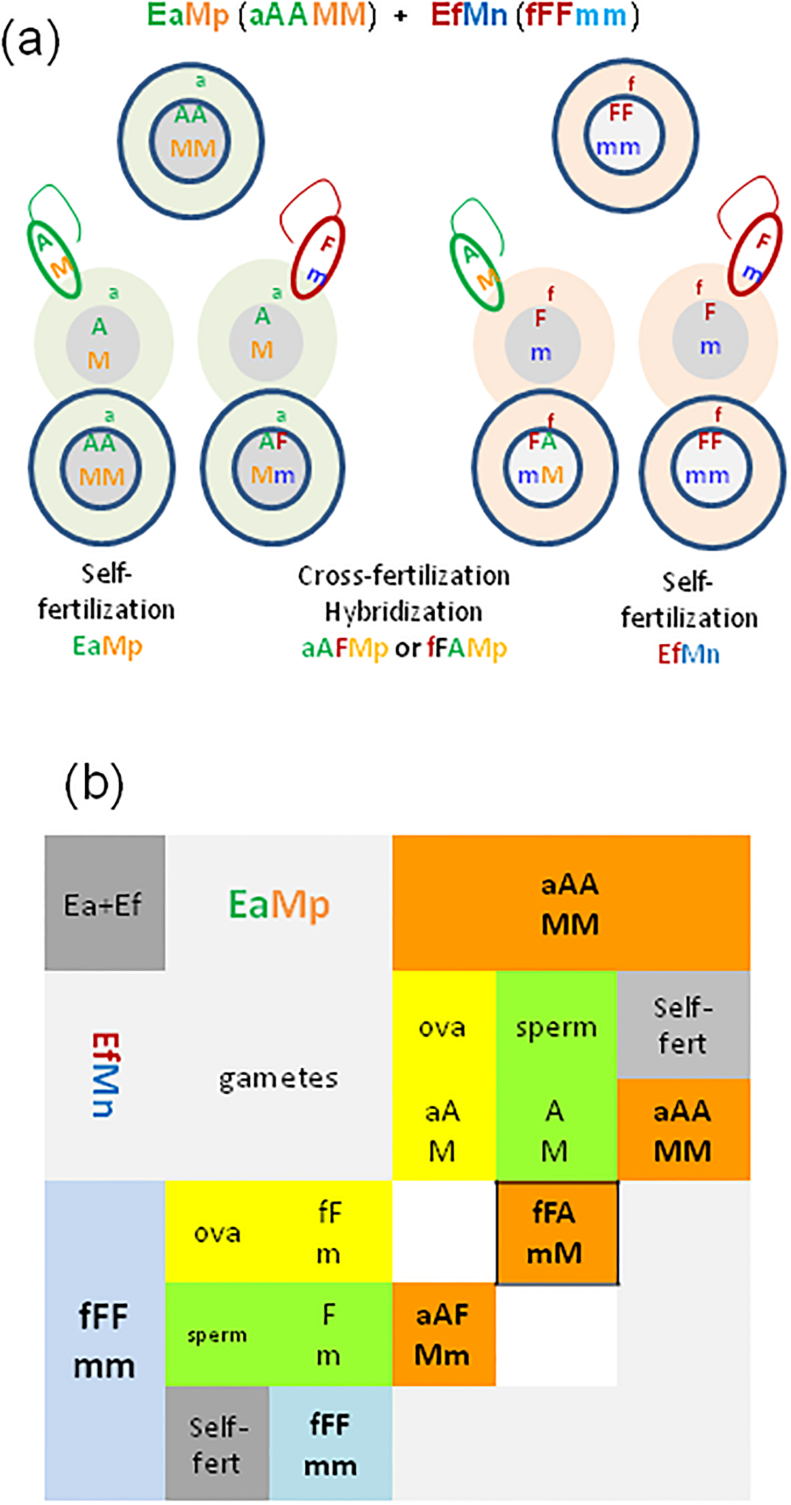
Hypothetical genotypes of Ea (aAAMM) and Ef (fFFmm) pairs and their offspring. a) Scheme of parental cells, their gametes (ova, spermatozoa) and zygotes, and b) the Punnett square. Assumption is that M-fluorescence might be encoded/controlled by the nuclear gene with the dominant ‘M’ allele and the recessive ‘m’ allele segregating independently from the nuclear A/F sequences of 28s rRNA gene. The ‘MM’ and ‘Mm/mM’ determines the M-positive (Mp) phenotype (in orange) while ‘mm’ genotype determines the M-negative (Mn) phenotype (in blue). Punnett squares are adapted to pairs of hermaphroditic earthworms able to self-fertilization; ova in yellow, spermatozoa in green. In each pair the first allele is that of maternal origin. Framed genotypes were apparently absent among investigated earthworms. Ova and resulted offspring with mito-nuclear incompatibility are crossed out.

As illustrated on Fig 3, during hybridization experiments starting with Ea+Ef pairs, the EaMp specimen of aAAMM genotype shall produce only one type of ova, i.e. aAM, and one kind of spermatozoa, AM. The Ef specimens, fFFmm, shall produce only fFm ova and Fm spermatozoa. The aAM ova may be either self-fertilized by AM spermatozoa giving aAAMM specimens or cross-fertilized by Fm spermatozoa of Ef partner giving the M-positive aAFMm hybrid. The fFm ova of fFFmm partner may be self-fertilized by Fm spermatozoa giving fFFmm M-negative Ef earthworms or by the AM spermatozoa of the Ea partner giving M-positive fFAmM hybrids (Fig 3a and 3b). However, fFAmM hybrids from Ef ova were absent among investigated specimens (framed in Fig 3b), that pointed out on asymmetrical hybridization of Ea and Ef, with hybrids derived preferentially (or exclusively) from the Ea ova.

Theoretically, the M-positive aAFMm hybrids might produce four types of oocytes, aAM, aAm, aFM, and aFm, the two latter genotypes less probable due to mitochondrial-nuclear (aF) incompatibility [20–22], and four types of spermatozoa, AM, Am, FM, and Fm. One could expect any possible combination resulting from self-fertilization of hybrids (see S1 Fig). However, the M-negative aAAmm and aAFmm, which theoretical might result from self-fertilized hybrid ova by any of hybrid sperm, were absent among 46 investigated earthworms. Moreover, according to our previous work, pairs of hybrids gave no viable offspring [18]. Nevertheless we cannot exclude of participation of hybrid self-fertilization during mating of aAF hybrids with parental species.

**Fig 4:**
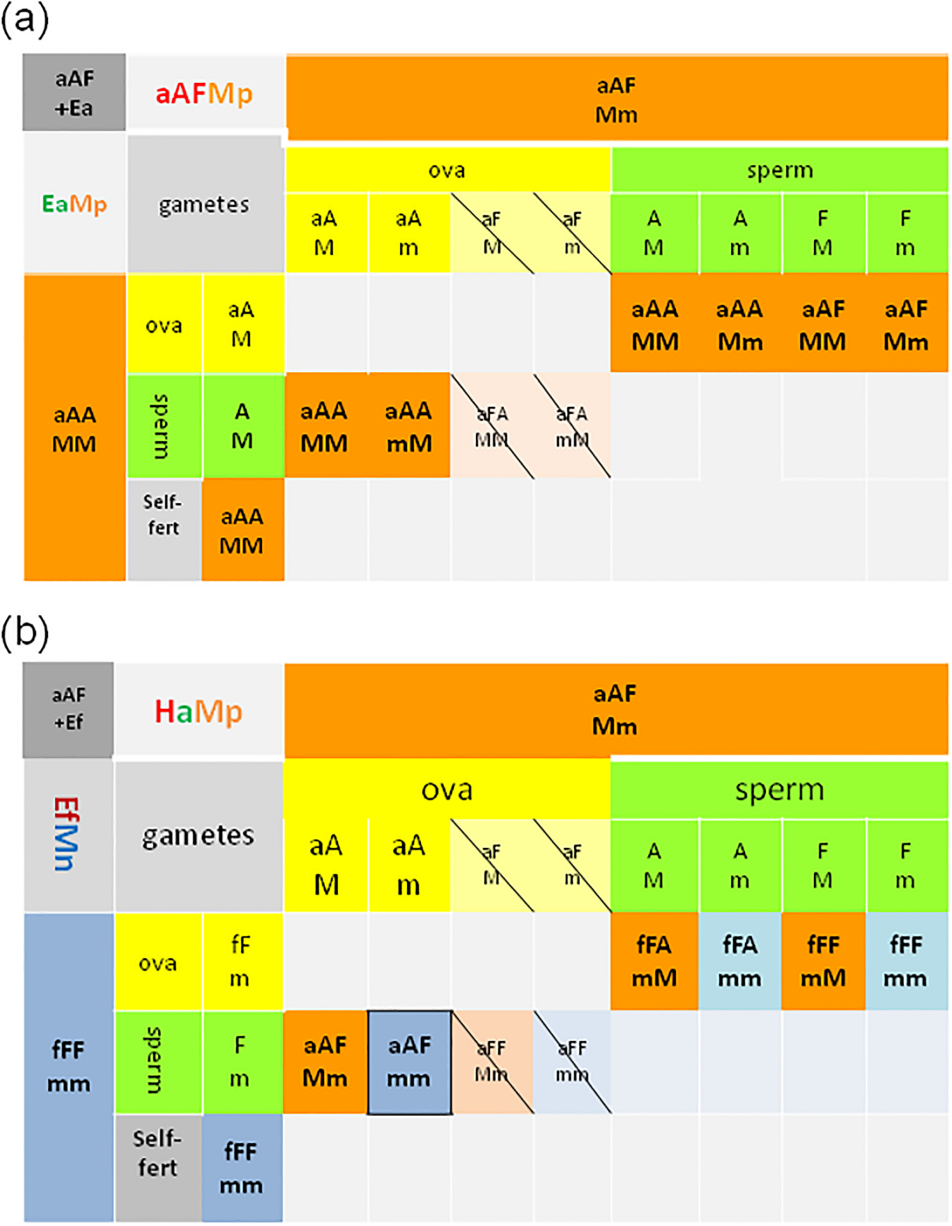
Hypothetical genotypes of the offspring of test-crosses of the hybrids (aAFMm) with parental specimens. Punnett squares of a) (aAFMm + aAAMM); b) (aAFMm + fFFmm) pairs. Self-fertilization within aAFMm hybrid is shown on S1 Fig thus is omitted here. Symbols are the same as on Fig 3.

The aAFMm hybrids gave a progeny in pairs with Ea or Ef specimens illustrated on Fig 4, where progeny from mito-nuclear incompatible ova are excluded, and aAF self-fertilization (shown on S1 Fig) is omitted for a clarity.

Only M-positive offspring (aAAMM; aAAMm/aAAmM) appeared in the aAFMm+aAAMM pairs, that was consistent with data on Fig 4a.

The offspring of aAFMm+fFFmm pairs from the hybrid’s ova (aAM and aAm), excluding those with mito-nuclear-incompatibility (aFM and aFm), might give both M-positive (aAFMm) and M-negative (aAFmm) hybrids (Fig 4b), but the latter were absent among investigated earthworms. The offspring from the Ef ova (fFm) consisted of both M-positive and M-negative hybrids (fFAmM and fFAmm), the ‘atypical’ M-positive Ef specimen fFFmM, and most common M-negative Ef (fFFmm) earthworm (Fig 4b).

**Fig 5:**
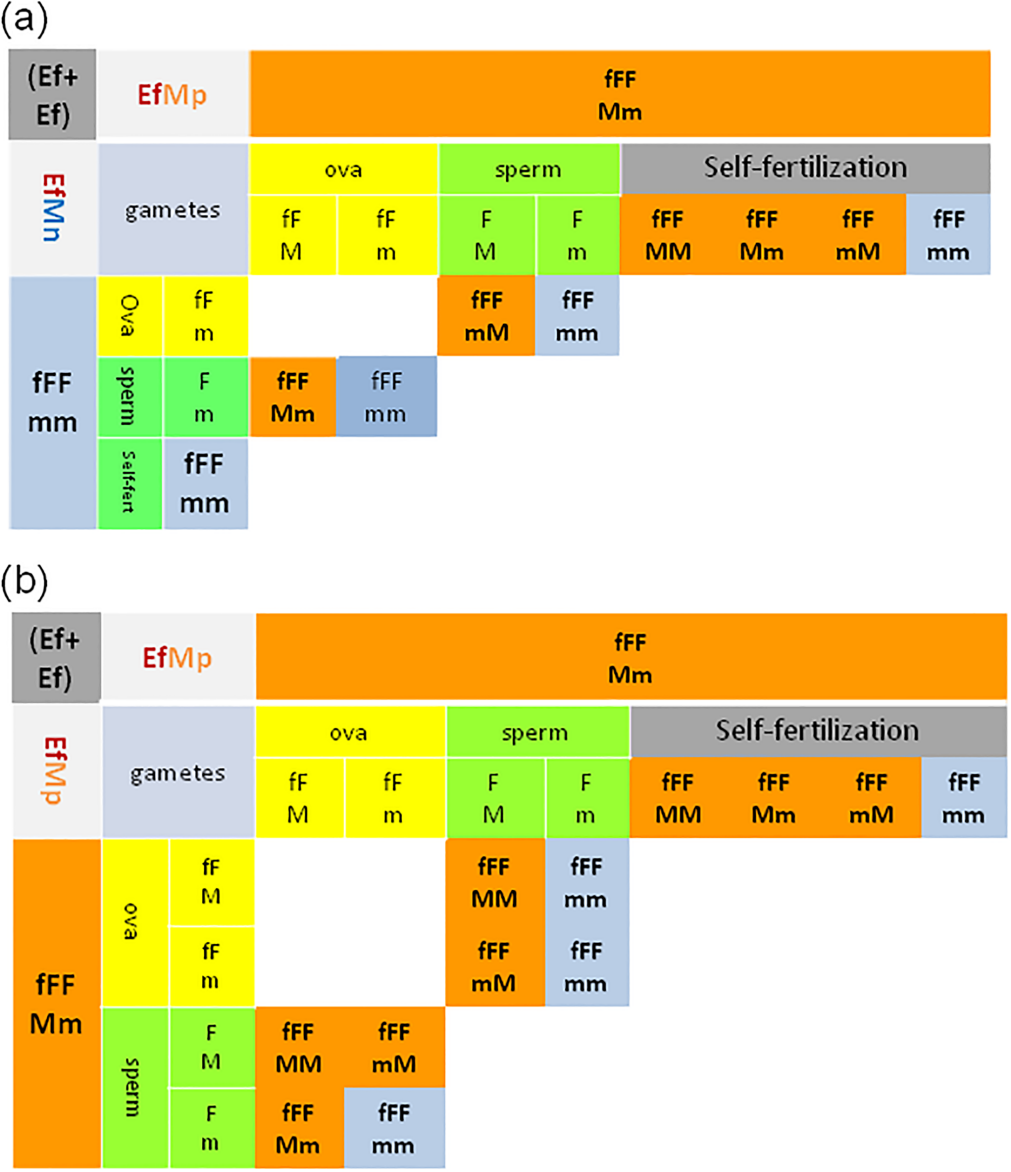
Hypothetical genotypes of the offspring of M-positive Ef specimens within EfMp culture. Punnett squares of a) (fFFMn+fFFMn) pair; self-fertilization shown in one partner only; b) (fFFMn+fFFmm) pair. Symbols are the same as on Fig 3.

Fig 5 shows that even one unique M-positive fFFMm specimen might initiate propagation of M-positive phenotype in ‘traditional’ Ef (fFFmm) culture; fFFMn/fFFMM genotypes might appear by self-fertilization and cross-fertilization with a ‘typical’ M-negative fFFmm partner (Fig 5a), and then by mating with newly-created other fFFMn earthworms (Fig 5b). On the other hand, Fig 5b illustrates how in the progeny of phenotypically ‘atypical’ M-positive Ef earthworms might reappear the ‘typical’ M-negative Ef specimens, that has happened in earthworms used for our proof-of concept investigations (see below).

### Proof-of concept investigations: Reappearance of M-negative Ef specimens among descendants of ‘atypical’ M-positive Ef earthworms

Earthworms from France were tested for presence/absence of MUF-fluorophore in 2013 [10] and then groups of them were cultured further separately as EaMp, EfMn, and ‘atypical’ EfMn specimens. Four years later, among progeny of EaMp and EfMn there were exclusively the M-positive Ea and M-negative Ef specimens, respectively. Among randomly sampled 7 specimens from the descendants of ‘atypical’ EfMp earthworms there were five M-positive (fFFMm) and two M-negative specimens (fFFmm). The results of our proof-of-concept investigations were consistent with hypothesis about inheritance of undefined gene with the dominant M-allele responsible for M-fluorophore in *Eisenia* sp. (Fig 5b).

### Pigmentation patterns of genotyped M-positive and M-negative earthworms

**Fig 6:**
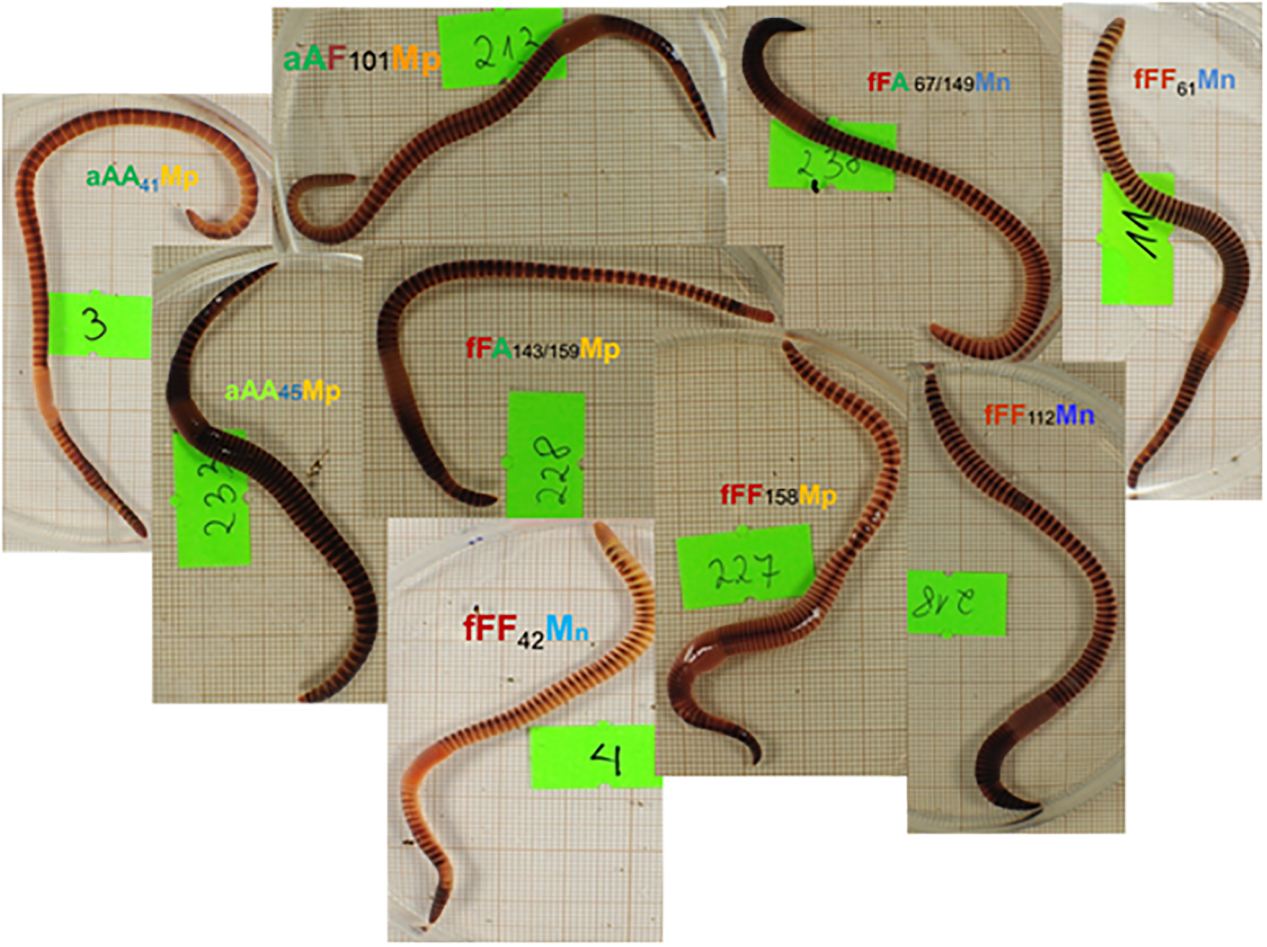
Pigmentation patterns of Ea, Ef and their hybrids with and without the M-fluorophore. Photos of M-positive (Mp) and M-negative (Mn) coded specimens of *Eisenia andrei* (aAA), *E*. *fetida* (fFF) and their hybrids (aAF and fFA). Symbols are the same as on Figs 1 and 2.

As shown on photos in Fig 6, the presence/absence of M-fluorophore did not correspond with pigmentation pattern of investigated earthworms. In general, inter-segmental grooves were hardly visible in relatively uniformly colored, lighter or darker, M-positive Ea specimens (aAA41Mp and aAA45Mp, respectively). Inter-specific hybrids, both M-positive aAF101Mp and fFA143/159Mp, and M-negative fFA67/149Mn, had slightly banded posterior parts of the body. Banding was distinct in Ef specimens, both M-positive (fFF158Mp) and M-negative (fFF42Mn, fFF61Mn, and fFF112Mn), with lighter or darker coloration and sharply demarcated much lighter inter-segmental grooves (Fig 6).

## Discussion

**Fig 7:**
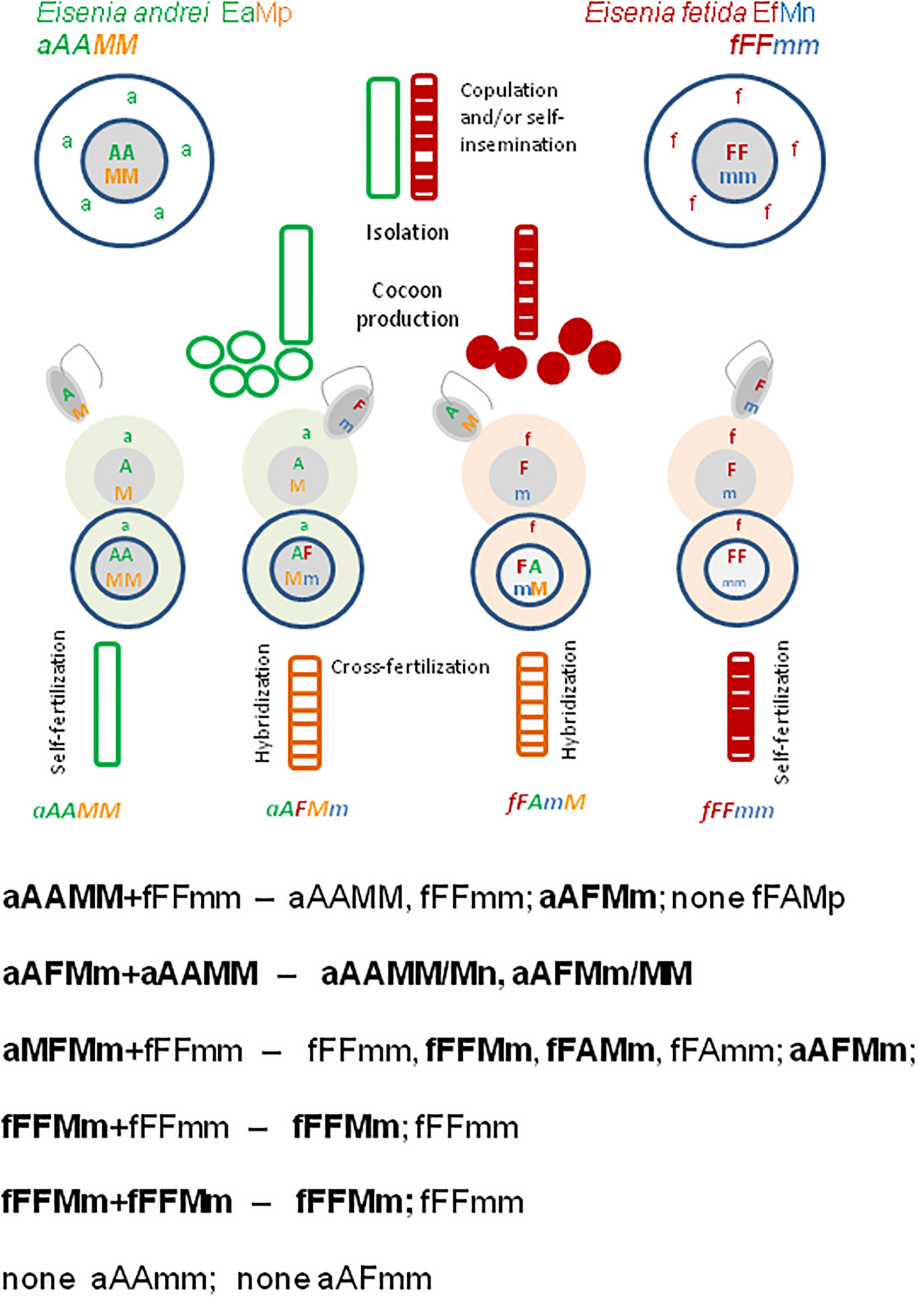
Scheme of mating and main results concerning Mp/Mn phenotypes of Ea, Ef and their hybrids. Combined summary of previous [18] and present experiments. Symbols are the same as on Fig 3.

Asymmetrical hybridization between Ea and Ef resulted in a wide spectrum of new phenotypes, including Ea-like earthworms with Ef-like banded posterior body parts, and the existence of Ea-specific M-fluorophore in coelomic fluid of most hybrids and some Ef earthworms; in other words, hybridization enriched the genetic pool of both species (Fig 7).

Adaptive value of uniform or banded body pigmentation patterns might be experimentally tested, e.g. through measurements of attractiveness for potential predators. So far, we may conclude that pigmentation patterns might be relevant for preliminary species/hybrid delimitation, and do not correspond with presence/absence of M-fluorophore in coelomic fluid. The M-fluorophore was considered as molecular marker of *E*. *andrei* while it turned out that is also present in majority of hybrids and some specimens of *E*. *fetida*. Thus, hybridization of Ea with Ef resulted in bi-directional gene flow; Ef-specific genes/alleles responsible for striped pigmentation are transferred from Ef to Ea. In contrast, hypothetical dominant M alleles of gene/s responsible for metabolic pathway leading to production of M-fluorophore flow from M-positive Ea, through M-positive Ea-derived hybrids to Ef, resulting in some M-positive Ef specimens. Even one M-positive Ef earthworm mated with M-negative partner propagates the M alleles within Ef culture; among offspring of ‘atypical’ M-positive Ef, the typical M-negative Ef specimens may reappear (Fig 7).

The M-negative Ea and M-negative Ea-derived hybrids were absent among 46 investigated earthworms although such phenotypes/genotypes are theoretically possible; they might be detected if the number of investigated earthworms would be increased. On the other hand, their viability might be impaired if the M-factor plays an important biological role in *Eisenia* species.

Better viability and higher fecundity of *E*. *andrei* than those of *E*. *fetida* were described by several scientific teams [23–25] including ours [18]. The same concerns Ea-ova derived hybrids that are fertile, in contrast to rare and sterile hybrids of Ef-ova origin. Thus, the question appears whether it is this dependent on the presence of M-fluorophore or some undiscovered metabolic pathways leading to its production?

Characteristic fluorescence spectra of coelomic fluid of *E*. *andrei* and *E*. *fetida* were for the first time used as specific fingerprints for taxonomy of these species in 2003, and authors stated that the unique fluorescence properties of *E*. *andrei* molecular marker are characteristic for the 4-methylumbelliferyl β-D-glucoronide (MUGlcU) [11], called here the M-fluorophore. In fact, fluorescence spectra similar to M-fluorophore have been shown in 2008 as those derived from methanol solution of 4-methyl umbelliferone, a member of coumarin family Coumarins are natural products present in ethereal oils of many plants, e.g. cinnamon (*Cinnamonum zeylanicum*) [26]. Biological effects of natural and synthetic coumarin derivatives include anti-plasmodial and antimalarian [27], anti-fungal [28], anti-tuberculosis [29], anti-coagulant [30], and anti-cancer [31] activities. In 2017, another aromatic metabolite unique for coelomic fluid of *E*. *andrei* was identified as the compound SP-8203, consisting two quinazoline-2,4-diones joined by an N-acetylspermine linker but its fluorescence spectra have not been analyzed in this paper [32]. Compound SP-8203 is pharmacologically potent in mammalian cells showing neuroprotective activity [33; 34]. The precise chemical characteristic of the M-fluorophore requires further analysis but that is not our current concern. Nevertheless, due to its hypothetical connections with pharmacologically potent factors, we may assume that the M-fluorophore might be somehow responsible for higher viability of M-positive *E*. *andrei* and M-positive Ea-derived hybrids than M-negative *E*. *fetida* and rare infertile Ef-ova derived hybrids. Further studies on the selected M-positive *E*. *fetida* might be fruitful in testing such supposition.

Speculations on hypothetical gene with the dominant M-allele are consistent with assumption of the intrinsic origin of M-fluorophore, being entirely dependent on the earthworm own metabolic pathways. Keeping in mind the peculiar copulatory behavior of lumbricid earthworms (Fig 7), hypothesis of microbial origin of M-fluorescence cannot be neglected. Almost all lumbricid earthworms harbor extracellular species-specific bacterial symbionts of the genus *Verminephrobacter* localized in their excetory nephridia [35; 36]. These symbionts are vertically transmitted via the cocoons containing developing embryos and persist in specific location throughout the whole lifespan of colonized earthworms [37; 38]. Recently it has been shown that bacterial symbionts have beneficial effects on maturation and reproduction of *E*. *andrei* [39]. Some products of bacterial metabolisms, including hypothetical M-fluorophore, might accumulate in earthworm coelomic fluid. Hypothetically, some of these extracellular symbionts may be released during copulation to the seminal fluid, and may reach spermathecas of the partners of copulation. Then they are released to cocoons together with sperm, and within cocoons infect ova or developing embryos resulted from self- or cross-fertilization. In such cases not only Ea and Ea-derived hybrids but also some Ef and Ef-derived embryos can be infected and became M-positive adults. In conclusion, the M-positivity of some earthworms might be considered as a result of ‘sexually-derived infection’ by some bacterial symbionts specific for *E*. *andrei*, responsible for metabolic pathways leading to production of M-fluorophore. It is also possible that both earthworm-derived and bacteria-derived factors must cooperate to give the final fluorescent product, that is either accumulated breakdown product being significant only as molecular biomarker, or may have unrecognized yet crucial biological significance.

On the basis of our previous results we may assume that coelomocytes are not the main cellular source of M-fluorophore in *E*. *andrei*, as its amount came back rapidly to the initial level after experimental expulsion of coelomic fluid [13, 19]. This makes M-fluorescence a reliable molecular marker for tracking the M-positivity among specimens of *E*.*andrei/E*.*fetida* complex, but other techniques shall be used to show conclusively its presence in various earthworm cell types lining coelomic cavity and/or other (bacterial?) sources.

## Conclusion

Asymmetrical hybridization between Ea and Ef resulted in bi-directional gene flow resulting in two phenomena recognized in our laboratory. First, Ef-like body pigmentation pattern appeared in posterior body segments of hybrids, both Ef- and Ea-derived; second, Ea-specific M-fluorophore was transferred to majority of hybrids and some Ef earthworms. The chemical nature and biological significance of this fluorophore is still an open question, but its fluorescence spectra are reliable markers for tracking the gene flow between *E*. *andrei* and *E*. *fetida*. If M-fluorophore is genetically controlled by hypothetical gene of *Eisenia* sp. with the dominant M allele, then such allele may be inherited by Ea-derived hybrid from M-positive Ea parent, and then transferred during mating with M-negative Ef earthworm into some *E*. *fetida* and some Ef-derived hybrids. Even one ‘atypical’ M-positive Ef might propagate this allele by crossing with ‘typical’ M-negative Ef. Vice versa, in cultures of M-positive Ef earthworms might reappear ‘typical’ M-negative specimens. However, hypothesis of the microbial origin of F-fluorescence derived from *E*. *andrei* specific bacterial symbionts cannot be neglected. Moreover, both the intrinsic and external factors might cooperate to produce the M-fluorophore. The existence of Ea and Ef hybridization make these common species easily maintained in laboratory the attractive models for studies on interspecies gene flow, inter-specific transmission of bacterial symbionts, and hypothetical effects of external factors on these phenomena.

## Supporting information

S1 Fig Hypothetical genotypes of the offspring of self-fertilizing hybrid (aAFMm) earthworm. a) Scheme of aAFMm parental cell, gametes (ova, spermatozoa) and zygotes; b) Punnett square. Shadowed parts of part ‘a’ and crossed out parts of part ‘b’ indicate mitochondrial-nuclear conflicts. Framed genotypes were absent among investigated earthworms. Assumption is that M-fluorescence might be encoded/controlled by the nuclear gene with the dominant ‘M’ allele and the recessive ‘m’ allele segregating independently from the nuclear A/F sequences of 28s rRNA gene. The ‘MM’ and ‘Mm/mM’ determines the M-positive (Mp) phenotype (in orange) while ‘mm’ genotype determines the M-negative (Mn) phenotype (in blue). Punnett square is adapted to pairs of hermaphroditic earthworms able to self-fertilization; ova in yellow, spermatozoa in green. In each pair the first allele is that of maternal origin. Framed genotypes were apparently absent among investigated earthworms. Ova and resulted offspring with mito-nuclear incompatibility are crossed out.
(TIF)
