# Supplementary figures and images for: Asymmetrical hybridization and gene flow between Eisenia andrei and E. fetida lumbricid earthworms
Source: PLoS One. 2018 Sep 21;13(9):e0204469. doi: 10.1371/journal.pone.0204469 (PMC6150523; doi:10.1371/journal.pone.0204469)

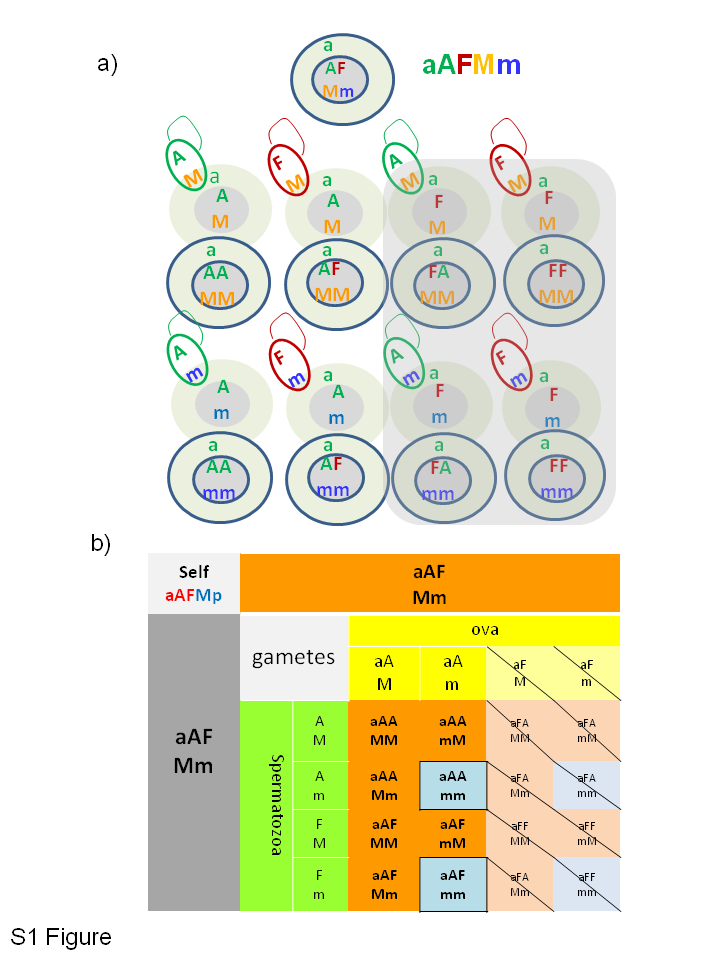

Supplement: S1 Fig — a) Scheme of aAFMm parental cell, gametes (ova, spermatozoa) and zygotes; b) Punnett square. Shadowed parts of part ‘a’ and crossed out parts of part ‘b’ indicate mitochondrial-nuclear conflicts. Framed genotypes were absent among investigated earthworms. Assumption is that M-fluorescence might be encoded/controlled by the nuclear gene with the dominant ‘M’ allele and the recessive ‘m’ allele segregating independently from the nuclear A/F sequences of 28s rRNA gene. The ‘MM’ and ‘Mm/mM’ determines the M-positive (Mp) phenotype (in orange) while ‘mm’ genotype determines the M-negative (Mn) phenotype (in blue). Punnett square is adapted to pairs of hermaphroditic earthworms able to self-fertilization; ova in yellow, spermatozoa in green. In each pair the first allele is that of maternal origin. Framed genotypes were apparently absent among investigated earthworms. Ova and resulted offspring with mito-nuclear incompatibility are crossed out. (TIF) [file pone.0204469.s001.TIF]
